# Effect of Blocking of Neuropeptide Y Y2 Receptor on Tumor Angiogenesis and Progression in Normal and Diet-Induced Obese C57BL/6 Mice

**DOI:** 10.5539/gjhs.v7n7p69

**Published:** 2015-03-26

**Authors:** Masoud Alasvand, Bahman Rashidi, S. H. Javanmard, Maziar Mohammad Akhavan, Majid Khazaei

**Affiliations:** 1 Department of Physiology, Isfahan University of Medical Sciences, Isfahan, Iran; 2 Department of Anatomy, Isfahan University of Medical Sciences, Isfahan, Iran; 3 Applied Physiology research center, Isfahan University of Medical Sciences, Isfahan, Iran; 4 Skin Research Center, Shahid Beheshti University of Medical Sciences, Tehran, Iran; 5 Department of Physiology, Faculty of Medicine, Mashhad University of Medical Sciences, Mashhad, Iran

**Keywords:** angiogenesis, melanoma tumor, nitric oxide, obesity, vascular endothelial growth factor

## Abstract

**Background:**

Obesity is a risk factor for some types of cancers. Angiogenesis is a necessary step in the multistage progression of tumors such as melanoma. Previous studies reported that neuropeptide Y (NPY) regulates angiogenesis by activating the Y2 receptor on endothelial cells. The present study examined the effects of the NPY Y2 receptor antagonist on tumor weight, angiogenesis and serum levels of vascular endothelial growth factor (VEGF), VEGF receptor-1 (VEGF-R1), and nitric oxide (NO).

**Methods:**

Twenty four male C57BL/6 mice were divided into control and obese groups. The control group was fed a normal diet whereas the obese group was fed a high fat diet. After 16 weeks, 2 × 10^6^ B16F10 melanoma cells were injected subcutaneously into all animals. Half of the control and the obese animals received 1 µM, 100 µL/kg NPY Y2 receptor antagonist (BIIE 0246) intraperitoneally. After two weeks, the animals were sacrificed, and angiogenic factors and tumor weights and angiogenesis were analyzed.

**Results:**

Tumor weight in the obese mice was higher than in the control (p<0.05). Treatment with BIIE 0246 reduced tumor weight in the obese animals (p<0.05), without effect on control group (p>0.05). Administration of an NPY Y2 receptor antagonist decreased tumor angiogenesis (evaluated as capillary density/mm^2^) and serum VEGF concentration in the obese group without altering serum VEGF-R1 and NO concentrations.

**Conclusions:**

Blockade of the NPY Y2 receptor suppressed tumor growth in obese mice by affecting tumor angiogenesis. Thus, it seems that NPY and its Y2 receptor antagonist might be new targets in melanoma tumor therapy.

## 1. Introduction

Obesity develops from an imbalance between energy intake and energy expenditure. Numerous health problems have been related to obesity (Pi-Sunyer, 2002). It is a risk factor for some types of cancer, such as breast, prostate, and colon cancers (Ogden, Carroll, McDowell, & Flegal, 2007). In the previous decade, several studies reported the link between melanomas and obesity (Mantzoros et al., 2007; Gogas et al., 2008; Renehan, Tyson, Egger, Heller, & Zwahlen, 2008). Melanoma is one of the most aggressive forms of skin cancer characterized by the malignant proliferation of melanocytes (Hayat, Howlader, Reichman, & Edwards, 2007). Research has shown that obesity caused by a high-fat diet stimulates melanoma metastasis in vivo (Jung et al., 2015). Angiogenesis is a necessary step in the multistage progression of malignant melanoma. The onset of new blood vessel formation is ushered by the release of VEGF and numerous other angiogenic molecules by the tumor cells (Schmieder et al., 2005). Depending on the tumor type, VEGF and its receptors may function via either the autocrine or paracrine mechanisms in humans (Cherrington, Strawn, & Shawver, 2000). One of the tyrosine kinase receptors for VEGF is VEGFR-1, and unlike fibroblast growth factor receptors this receptor is selectively expressed in endothelial cells (ECs) (Friesel & Maciag, 1955). The primary role of VEGFR-1 is to regulate the assembly of endothelial cells (ECs) into tubes, whereas VEGFR-2 prompts permeability and ECs differentiation and proliferation (Ellis, Takahashi, Liu, & Shaheen, 2000). Since the raised expression of VEGF and its receptors is closely correlated with tumor vascularity, progression, and metastasis, targeting VEGF/VEGFRs becomes a useful strategy (Ivy, Wick, & Kaufman, 2009; Takahashi, Kitadai, Bucana, Cleary, & Ellis, 1995).

Nitric oxide (NO) is an important bioactive material and signaling molecule that mediates a variety of biological actions such as vasodilatation, neurotransmission, and host defense. It has been proposed to contribute to the pathogenesis of cancer (Geller & Billiar, 1998). It has also been found that NO is present in tumor tissues and that its level and persistence may affect tumor progression or repression (Mocellin, Bronte, & Nitti, 2007).

Neuropeptide Y (NPY) is a 36 amino-acid peptide that is produced at different locations including vascular endothelial cells. NPY regulates the cardiovascular system, feeding behavior and angiogenesis (Pedrazzini, Pralong, & Grouzmann, 2003). The effect of NPY via Y receptors can vary from tumor growth promotion to tumor growth inhibition and induction of apoptosis. NPY also promotes vascularization by activating the Y2 receptor on ECs, resulting in a continuous supply of nutrients to these fast growing tumors (Ekstrand et al., 2003). Using Y2 receptor antagonists could therefore be a strategy to treat tumors and, at the same time, may have beneficial effects against cancer induced by body weight (Zhang, Bijker, & Herzog, 2011). This study evaluated the effects of BIIB 0246 on the serum concentrations of VEGF, NO, and the soluble forms of VEGFR-1 as well as tumor capillary density and tumor weight in control and obese mice.

## 2. Material and Methods

### 2.1 Animal Experiments

Briefly, 24 male C57BL/6 mice, 4–5 weeks of age and weighing 20-30 g, were purchased from the Pasteur Institute of Tehran, Iran. Animals were housed in cages in animal facilities under the following conditions: temperature 25°C ± 2, lighting cycle of 12h (6:00 AM to 6:00 PM), and free access to food and water ad libitum. Animals were divided into four groups: O: obese, N: control (normal), O+AntaNPY (obese+NPY Y2 receptor antagonist) and N+AntaNPY (control+NPY Y2 receptor antagonist) (n=6 each) and body weight was measured once a week. The control group was fed a normal diet whereas the obese group was fed a high fat diet (HFD; BioServ Co, Cat #F3282, USA) (Peyot, 2010). Both groups of mice were fed for 16 weeks. Half of the control and obese animals received NPY Y2 receptor antagonist (BIIB 0246). NPY Y2 receptor antagonist was obtained from Tocris Co. (Bristol, UK) and 1µM, 100 μL/kg, was injected intraperitoneally (Pandey, Vijayakumar, Ajay, Malvi, & Bhat, 2012).

### 2.2 Cell Culture

B16F10 melanoma cells which can grow in the C57BL/6 strain mouse were purchased from the National Cell Bank of Iran (NCBI, Pasteur Institute of Iran). Cells were cultured in DMEM supplemented with 4 mM L-glutamine, 4.5 g/l glucose, 10% FBS, and antibiotics (100 μg/ml streptomycin, 100 μg/ml penicillin) under humidified air with 5% CO2 at 37°C. After 80% confluence of the melanoma cell monolayer in culture, the cells were washed and detached with PBS containing 0.25% trypsin and 0.03% EDTA and then pelleted by brief centrifugation at 100 g. The supernatant was removed, cell pellets were suspended in PBS, and the cell number was counted.

### 2.3 Tumor Challenge

C57BL/6 mice were inoculated subcutaneously with 2×106 B16F10 melanoma cells in the right flank, and tumors were induced with the injection of these cells. Mice were observed for the initiation and progression of melanoma tumors. Once the tumors became palpable, measurements were taken every day. Primary palpable tumors established on day 7-8. On day 8, the tumor-bearing mice were randomly dispensed into 4 groups, each containing 6 mice. Two groups received daily intraperitoneal (i.p.) injections of normal saline, and two groups received i.p. injections of NPY Y2 receptor antagonist. Tumor volume was calculated using the formula: Tumor volume=0.52 × a × b^2^ (a-longest diameter and b-shortest diameter). Tumor growth was followed up to 14 days after its initiation as tumors in normal and obese groups. 14 days after drug administration, the animals were sacrificed, and the tumors were then carefully dissected and weighed.

### 2.4 ELISA Assay of Serum VEGF

VEGF concentrations were defined in serum samples by enzyme-linked immunosorbent assay (ELISA) according to the manufacturer’s instructions (Quantikine; R&D Systems, Minneapolis, MN, USA). Briefly, this analyze employs the quantitative sandwich enzyme immunoassay technique with monoclonal antibodies, definite for VEGF pre-coated onto a microplate. Standard controls and samples were pipetted into the wells. After growth factor binding and washing, an enzyme-linked antibody specific for VEGF was added to each well. After washing, a substrate solution was added to the wells and color developed in proportion to the amount of growth factor bound in the first step. Optical density of each well was determined by a microplate reader at 450 nm. A blank was subtracted from both the standard controls and the samples. A standard curve was made by plotting the logarithm of the mean absorbance of each standard versus the logarithm of the soluble factor concentration. Concentrations are described as picograms per milliliter.

### 2.5 Serum VEGF-R1 Measurements

VEGFR1 concentrations were measured by enzyme-linked immunosorbent assay using available reagents and recombinant standards (R&D systems, Minneapolis, MN, USA).

### 2.6 Serum NO Measurement

Serum NO concentrations were determined using the Griess reagent method (Promega Corp, Madison, WI, USA) according to the manufacturer’s instruction.

### 2.7 Capillary Density Analysis

The animals were sacrificed at 14 days after treatment. Tumors were dissected, and capillary density was evaluated in the tumors by immunohistochemical staining. The samples were embedded in paraffin. Histological sections with 4 μm thickness were stained with monoclonal antibody directed against muse CD31 (Abcam, Cambridge, UK) (Li, 2008). Endothelial cells were recognized with CD31-positive cells and were counted by a light microscope. Ten microscopic fields (×400) from four different sections in each tissue block were randomly selected, and the number of capillaries was counted by two blinded observers. Capillary density was expressed as the number of capillaries per mm^2^.

### 2.8 Statistical Analysis

Values are expressed as means ± SE. Comparisons between groups were analyzed using analysis of variance (ANOVA) with a post hoc test, LSD. Significant difference was determined at a 0.05 probability level. All statistical analyses of data were performed using SPSS (version 16).

## 3. Results

### 3.1 Body Weight

**Figure 1:**
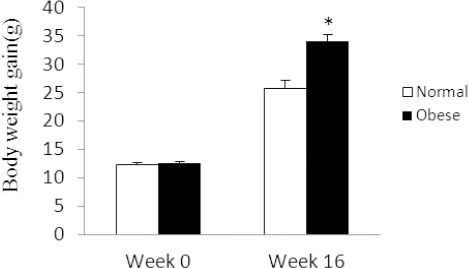
Body weight gain of mice in normal and obese groups Data is shown as mean ± SEM (n=6). *: P<0.05.

Results showed that the body weight of the mice fed a high fat diet was significantly higher than that of the mice fed normal chow (34±1.11 g vs. 25.66±1.41 g; p<0.05) (Figure 1).

### 3.2 Effects of NPY Y2 Receptor Antagonist on Tumor Weight

As shown in the Figure 2A tumor weight in obese mice on an HFD diet was significantly higher than that of control mice on a normal feed diet (6.47±0.79 vs. 3.48±1.1 g; p<0.05). Tumor weight in the obese group treated with BIIB 0246 was significantly less than that of non-treated groups (3.36±0.57 vs. 6.47±0.79 g; p<0.05), but BIIE 0246 did not have a significant effect on the control group (5.44±1.77 vs. 3.48±1.1 g; p>0.05). Images of the size of melanoma tumors in the obese and control groups are shown in Figure 2B.

**Figure 2A:**
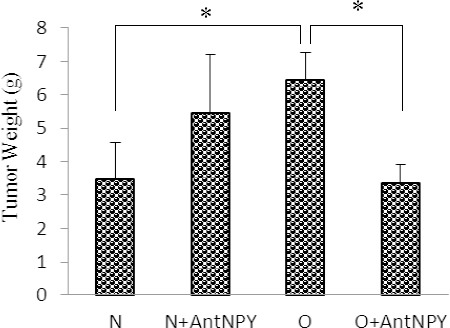
Tumor weight was significantly higher in obese mice than in control mice Administration of BIIE 0246 reduced tumor weight in the obese group compared with the non-treated group, but it had no significant effects on the control group. N: normal, O: obese, N+AntNPY: normal+NPY Y2 receptor antagonist. O+AntNPY: Obese+NPY Y2 receptor antagonist. Data is shown as mean ± SEM (n=6). *P<0.05.

**Figure 2B:**
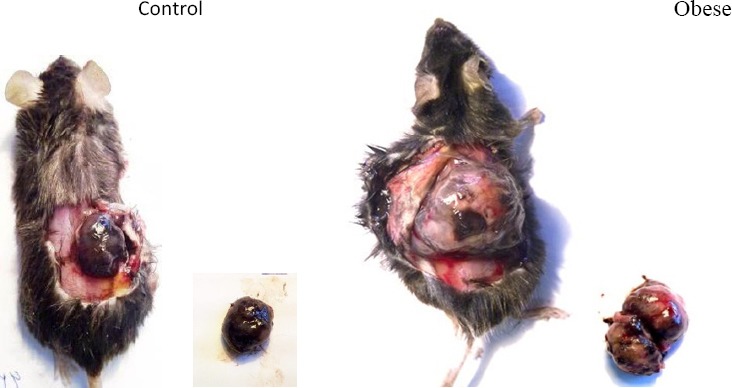
Images of melanoma tumor initiation and progression in control and obese mice, showing differences in weight and final size of melanoma tumors

### 3.3 Effects of NPY Y2 Receptor Antagonist on Serum VEGF Concentrations

**Figure 3:**
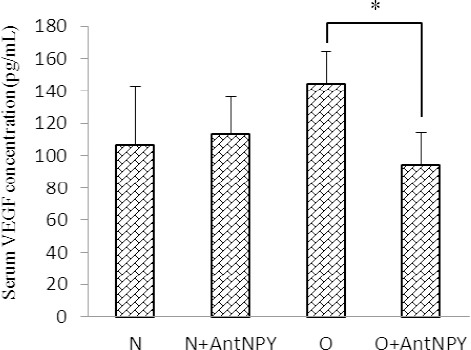
Administration of BIIE 0246 reduced the serum VEGF concentration in the obese group compared with the non-treated group, but did not significantly affect the control group N: normal, O: obese, N+AntNPY: normal+NPY Y2 receptor antagonist. O+AntNPY: obese+ NPY Y2 receptor antagonist. Data is shown as mean ± SEM (n=6). * P<0.05.

Figure 3 shows that the administration of BIIE 0246 reduced the serum VEGF concentration in the obese group compared with the non-treated group (144.3±20.4 vs. 94.3±20.1 pg/ml; p<0.05), but did not have a significant effect on the control group (106.3±36.6 vs. 112.9±23.6 pg/ml; p>0.05).

### 3.4 Effects of NPY Y2 Receptor Antagonist on Serum VEGF-R1 Concentrations

**Figure 4:**
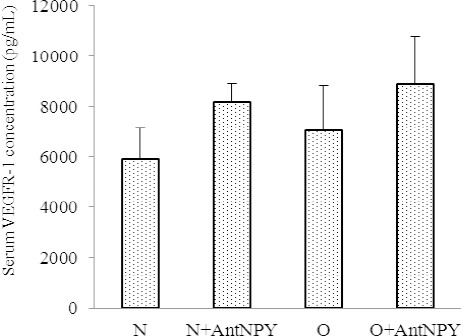
Administration of BII0246 had no significant effect on serum VEGF-R1 concentrations in either the control or the obese group N: normal, O: obese, N+AntNPY: normal+NPY Y2 receptor antagonist. O+AntNPY: obese+ NPY Y2 receptor antagonist. Data is shown as mean ± SEM (n=6).

Administration of BIIE 0246 had no significant effect on serum concentrations of VEGF-R1 in the control (8217.5±715.4 vs. 5933.5±1221.4 g/ml; p>0.05) or the obese group (8902.1±1884.6 vs. 7058.9±1796.1 Pg/ml; P>0.05) (Figure 4).

### 3.5 Effects of NPY Y2 Receptor Antagonist on Serum NO Concentrations

**Figure 5:**
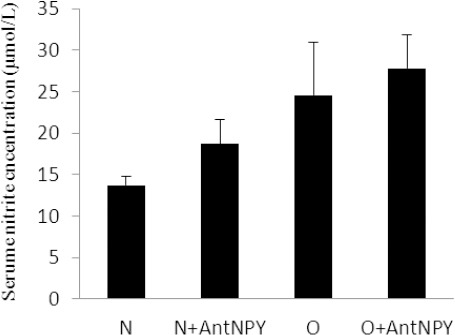
Administration of BIIE 0246 had no significant effect on serum NO concentrations in either the control or the obese group N: normal, O: obese, N+AntNPY: normal+NPY Y2 receptor antagonist. O+AntNPY: obese+ NPY Y2 receptor antagonist. Data is shown as mean ± SEM (n=6).

Measurement of serum NO level showed that there were no significant differences in serum NO concentrations between the control (13.7±1.1 vs. 18.7±2.9 µmol/L; p>0.05) and obese groups (24.6±6.3 vs. 27.8±4.1 µmol/L; P>0.05) (Figure 5).

### 3.6 Effects of NPY Y2 Receptor Antagonist on Capillary Density

We investigated capillary density (number of capillaries per mm^2^) in histological sections harvested from melanoma tumors in experimental groups. Administration of BIIE 0246 decreased capillary density in the control group compared with the control non-treated group (11.3±1.3 vs. 5.33±1.45 number/mm^2^; p<0.05). Also in the obese group, the capillary density in treated animals was significantly lower than in obese non-treated animals (8.5±1.55 vs. 4.66±0.55 number/mm^2^; P<0.05) (Figure 6A). Samples of immunohistochemical staining with anti-CD31 monoclonal antibody are shown in Figure 6B.

**Figure 6A:**
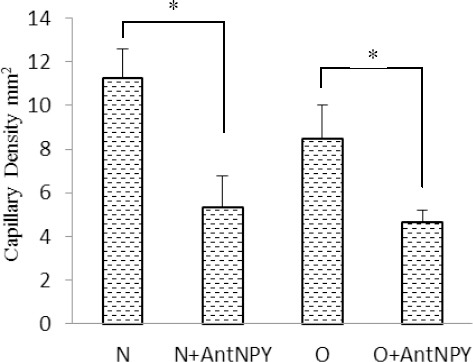


**Figure 6B:**
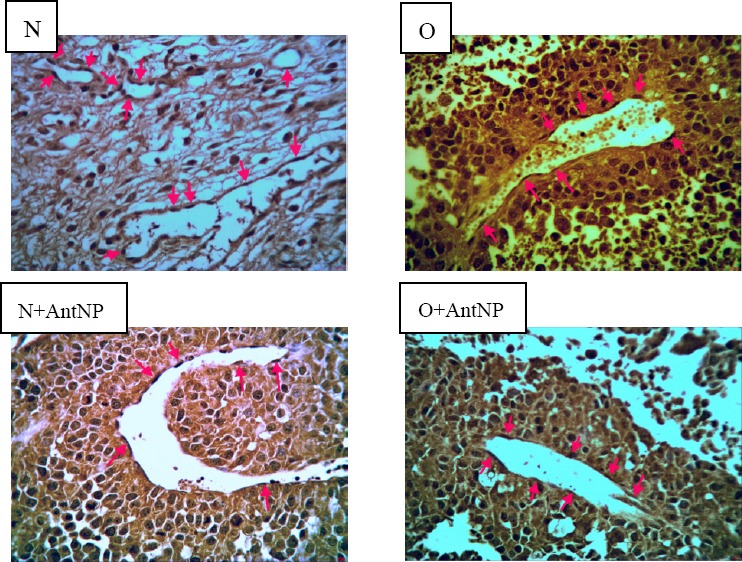


## 4. Discussion

In this study, the relationship between diet-induced obesity and melanoma progression and the effects of NPY Y2 receptor antagonist (BIIE 0246) on tumor angiogenesis and tumor progression in male C57BL/6 mice were investigated.

Obesity and melanoma morbidity have increased in recent years. Higher body weight has been related to increased melanoma occurrence in epidemiological studies (Renehan, Tyson, Egger, Heller, & Zwahlen, 2008; Dennis, Lowe, Lynch, & Alavanja, 2008). Investigators have recognized a large number of tumors sensitive to NPY mediated Y-receptor signaling. The effects of signaling via Y receptors can differ from tumor growth elevation to tumor growth inhibition and induction of apoptosis depending on the type of tumor tested. Therefore, agonists along with antagonists for Y receptors could be used to target cancers cells (Zhang, Bijker, & Herzog, 2011). An increase in NPY activity may be associated with cancer development via actions to promote weight gain, since overweight and obesity are related to an increased risk of cancer progression (Zhang et al., 2011).

In the present study, we found that diet-induced obesity caused increased tumor weight. This finding is in agreement with those of Vimal et al who showed that obesity-induced metabolic changes directly affect the transcriptional regulation of Cav-1, the protein expression of Cav-1, and FASN, being the main mediators of proliferation in melanoma cells (Pandey, Vijayakumar, Ajay, Malvi, & Bhat, 2012).

We also found that the levels of VEGF in the obese treatment group were lower than those in the non-treated group, while VEGFR-1 and NO serum concentrations had no significant differences between the control and obese animals. Results further showed that the capillary density in both the control and obese groups treated with BIIE 0246 were lower than those in the non-treated groups.

Angiogenesis is an important mediator in tumor progression. VEGF is one of the main cytokines that can stimulate angiogenesis and it promotes the proliferation of new blood vessels. There is also a clear correlation between intensity of VEGF and tumor prognosis (Roland et al., 2009). NPY seems to promote both the formation of collaterals and capillary angiogenesis. The Y2 receptor appears to be responsible for the angiogenic effects of NPY (Zukowska, Grant, & Lee, 2003; Kitlinska, Lee, Movafagh, Pons, & Zukowska, 2002; Ghersi, Chen, Lee, & Zukowska, 2001).

The potency of NPY in stimulating angiogenesis appears to be similar to the potency of VEGF (Zukowska et al., 2003). As in the case of VEGF which stimulates neovascularization partly by releasing NO, it appears that endothelial NO is essential for NPY’s angiogenic signaling, in part due to its upregulation of NPY receptor expression (Zukowska et al., 2003).

Results of the current study showed that BIIE 0246 could significantly decrease VEGF in treatment groups. Several experimental and clinical reports examining the effects of NPY Y2 receptor antagonist on serum levels of VEGF; some of these results are consistent with the findings regarding the VEGF levels reported in the current study. Congyi Lu et al showed that blocking the NPY Y2 receptor pathway by BIIE 0246 impaired tumor vascularization and enhanced apoptosis which, together with the direct effect on neuroblastoma cells, led to significant inhibition of tumor growth in vivo (Lu et al., 2010).

In the present study, although the obese treatment animals showed lower capillary density, their serum VEGFR-1 concentration had no significant difference with non-treated groups. It has been suggested that, even with an increase or no change in the VEGFR-1 level, the VEGFR-1 signaling pathway is defective during treatment, and these groups are probably VEGFR-1 resistant (Sasso et al., 2005; Waltenberger, 2009). It has also been suggested that high levels of VEGFR-1 inhibit angiogenesis by antagonizing the effects of VEGF (Ahmed, Li, Dunk, Whittle, Rushton, & Rollason, 1995; Maynard et al., 2003). This study could not demonstrate significant differences of NO levels in treatment groups compared with control groups. There is contradictory data about the role of NO in angiogenesis. Using an in vivo model, investigators have shown NO inhibits angiogenesis (Pipili-Synetos et al., 1994; Pipili-Synetos, Sakkoula, & Maragoudakis, 1993). Yang et al also reported that NO inhibits the proliferation of several different cell types, including vascular endothelial cells (Yang, Ando, Korenaga, Toyooka, & Kamiya, 1994). On the other hand, Joshi showed that NO originating from within the tumor cells may be responsible for the antiangiogenic effect within the tumor (Joshi, 1997). Regulation of blood flow by NO in experimental tumor-associated neovasculature has been reported (Tozer, Prise, & Chaplin, 1997) and may explain the vasodilation observed in the fully vascularized tumor. This conflicting data suggests that not only there is more than one mechanism involved, but also the type of tissue and tumor is an important factor in the angiogenic procedure. Thus, the role of NO in tumor angiogenesis needs further investigation.

## 5. Conclusion

The current study has shown that blocking the NPY Y2 receptor is beneficial to inhibiting tumor growth in vivo. The main mechanisms of this effect include inhibition of tumor cell proliferation with weakening of angiogenesis. The effectiveness of this treatment can be further improved by designing more potent NPY Y2 receptor antagonists. Therefore, NPY and its Y2 receptor are promising new targets in melanoma tumor therapy.
